# Topic Evolution Analysis for Omics Data Integration in Cancers

**DOI:** 10.3389/fcell.2021.631011

**Published:** 2021-04-07

**Authors:** Li Ning, He Huixin

**Affiliations:** ^1^Business School of Huaqiao University, Quan Zhou, China; ^2^Management Science and Engineering Department, Management School, Xiamen University, Xiamen, China; ^3^Computer Science and Engineering Department, Computer Science and Engineering School, Huaqiao University, Quanzhou, China

**Keywords:** omics, cancers, topic modeling, prophet neural network, evolution trend

## Abstract

One of the vital challenges for cancer diseases is efficient biomarkers monitoring formation and development are limited. Omics data integration plays a crucial role in the mining of biomarkers in the human condition. As the link between omics study on biomarkers discovery and cancer diseases is deepened, defining the principal technologies applied in the field is a must not only for the current period but also for the future. We utilize topic modeling to extract topics (or themes) as a probabilistic distribution of latent topics from the dataset. To predict the future trend of related cases, we utilize the Prophet neural network to perform a prediction correction model for existing topics. A total of 2,318 pieces of literature (from 2006 to 2020) were retrieved from MEDLINE with the query on “omics” and “cancer.” Our study found 20 topics covering current research types. The topic extraction results indicate that, with the rapid development of omics data integration research, multi-omics analysis (Topic 11) and genomics of colorectal cancer (Topic 10) have more studies reported last 15 years. From the topic prediction view, research findings in multi-omics data processing and novel biomarker discovery for cancer prediction (Topic 2, 3, 10, 11) will be heavily focused in the future. From the topic visuallization and evolution trends, metabolomics of breast cancer (Topic 9), pharmacogenomics (Topic 15), genome-guided therapy regimens (Topic 16), and microRNAs target genes (Topic 17) could have more rapidly developed in the study of cancer treatment effect and recurrence prediction.

## Introduction

Genomics, proteomics, metabolomics, transcriptomics, and other -omics studies involve comprehensive investigations (Saito et al., 2013). In recent years, advances in high-throughput technology have shown promise for discovering biomarkers (Njoku et al., 2020). Biomarkers are useful tools as indicators/predictors of disease severity and drug reactivity, and thus, are expected to be used for diagnostic or prognostic purposes for all different types of complex diseases. With the discovery and identification of HRAS and TP53, more proto-oncogenes, tumor suppressor genes, and susceptibility genes have been discovered (Hanahan and Weinberg, 2000, 2011). The essential characteristics of tumor cells have been elucidated at the molecular level. Combined with clinical information, the molecular mechanism and evolutionary dynamics of tumor development and cell heterogeneity can be observed (Urh and Kunej, 2016). Omics data integration and machine learning algorithm can be utilized to improve the predicting accuracy of familial tumor patients.

Meanwhile, biomarker discovery technology can also improve the sensitivity and accuracy of early diagnosis and provide more accurate molecular staging (Long et al., 2019). The combination of proteomics and metabolomics can reduce adverse reactions of targeted drugs and chemotherapy drugs (Ristori et al., 2020). Pharmacogenomics can be applied to detect new therapeutic targets and develop new drugs, and it can also turn old drugs into treasures (Shukla, 2017). Essential marks of metabolomics are biomarker development and its translation to the clinic that can do a favor to personalized diagnosis and deepen the understanding of disease pathogenesis (Nazifova-Tasinova et al., 2020). Despite the rapid development of omics data integration toward mining biomarkers in the academic medical world, few studies explored statistical relationships between literature text terms and their time-series features. Here, we present a topic modeling and predictive analysis for omics study in cancer diseases.

In recent years, scientific production on omics data integration toward the mining of phenotype biomarkers have produced datasets of significant extreme interests and has expanded the physiology field of cell and developmental biology concepts. Research in this area is indeed kinds of. But how is this bunch of research evolved? Which directions are more valuable for future development? Unfortunately, few studies explore the underlying relationships in existing reports among title, abstract, and keywords. Practically, two methods, natural language or bibliometric analyses, can achieve this goal. For one thing, employing natural language processing cuts sentences into metadata; for another thing, using the bibliometric method statistically analyzes metadata based on the time and frequency of occurrence. Intuitively, combining the two would possibly make a valuable prediction of potential hot spots from the technical perspective. An acceptable way to accomplish that goal is to evaluate the frequency of emerged scientific terms and how the same words are aggregated in research. Furthermore, Latent Dirichlet Allocation (LDA, henceforth), as a statistical technique, is available to capture and explain potential relationships between the high-frequently-used terms in recent scientific products with high precision through a layered aggregation system of words Hence, compared with traditional bibliometric research paper, this article is characterized by natural language processing of many title texts and predictive analysis based on time series. From this perspective, we summarize the scientific terms presented on MEDLINE over the past 15 years. Research topics are extracted, and then theme intensity is calculated based on the time segment of the month. From the perspective of topic modeling and the predictive correction algorithm optimization, our study provides specific methods for scientific researchers in the cell development field. It furnishes more followers with intuitive understanding and disciplinary analysis in the study of omics data integration to biomarkers.

## Methods

**Figure 1:**
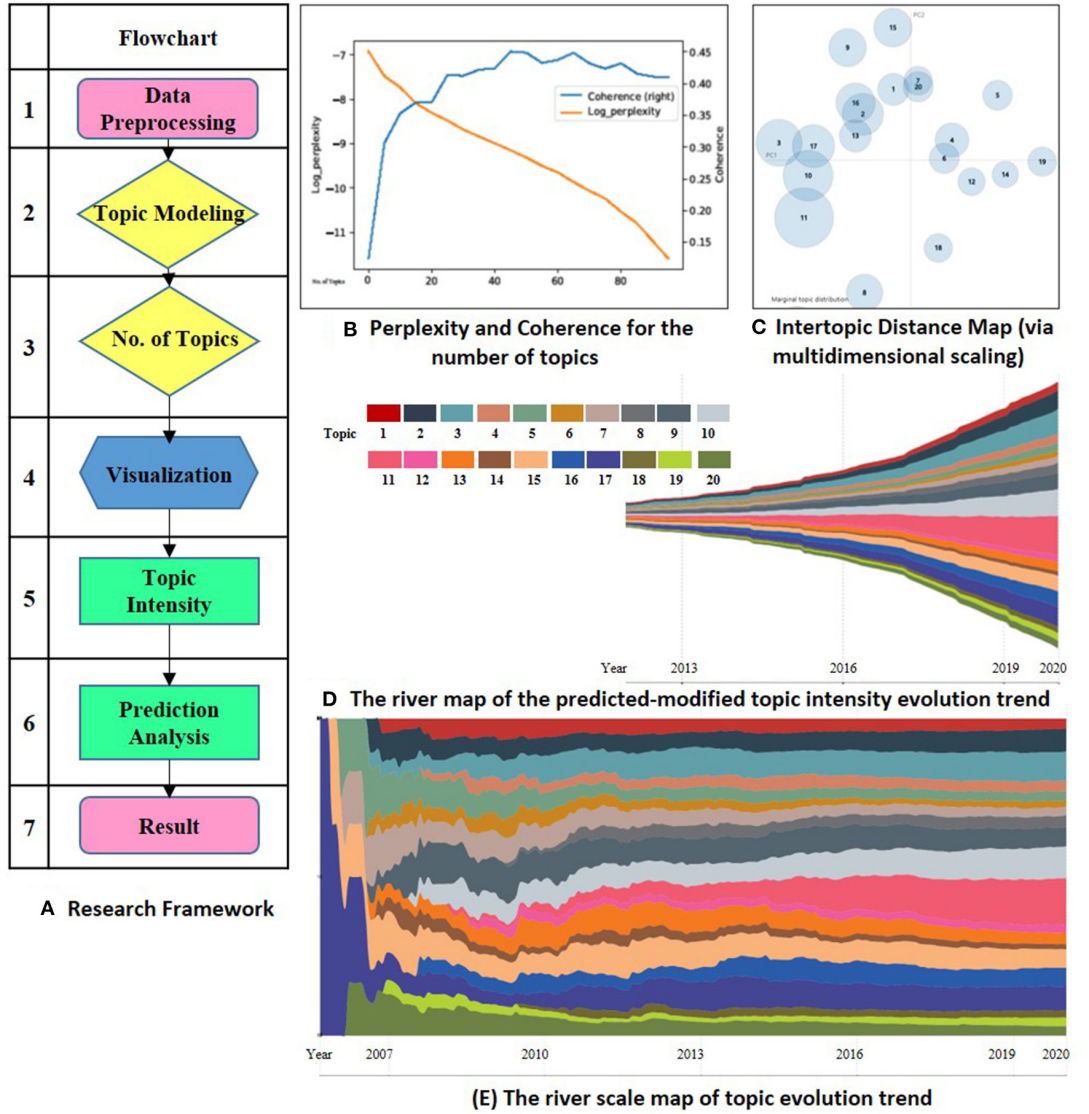
**(A)** is the research framework. **(B)** shows the perplexity and coherence for the number of topics. **(C)** visualizes the lntertopic distance for the 20 topics. **(D,E)** is the river map and river scale map of the predicted-modified evolution. Topic 1 biomarker discovery in early diagnostics, 2 machine learning for cancer prediction, 3 novel biomarker identify technology, 4 multi-omics in Hepatocellular Carcinoma, 5 genomics in clinical application, 6 genomics in tumor heterogeneity discovery, 7 proteomics in post-translational modification, 8 transcriptional and metabolic processes, 9 metabolomics of breast cancer, 10 genomics of colorectal cancer, 11 multi-omics analysis, 12 cancer vaccines, 13 tumor immunotherapy, 14 metabolomics in prostate cancer, 15 pharmacogenomics, 16 genome-guided therapy regimens, 17 non-coding RNA target genes, 18 microbial metabolomics, 19 metagenomics, 20 genomics of colorectal cancer.

This study evaluates the “Omics data integration” in “Cancers” to explore the research topic's evolution. The research framework contains seven steps, which are mainly embodied in two stages. The first phase is data preprocessing and topic extractions (Ning et al., 2020). Since the intensity of each subject has time-series features, so the second phase is the prediction analysis and display of the topic trend. Figure 1 shows the research framework in Panel (A).

Dataset for our study includes titles, abstracts, and keywords from publications. Data sources come from the Web of Knowledge-MEDLINE database. We used the following keywords to extract literature: omics [TS] AND cancer [TS] AND “2006/01/01 [DATE]: “2020/12/31 [DATE]”. 2,801 pieces of literature were obtained. Information retrieved includes title, author, abstract, keywords, references, and journal sources. We then filtered the literature to preserve only journal articles for downstream analysis, leaving 2,318 unduplicated items. All of the observed article titles, abstracts, and keywords were further processed by natural language routine.

### Topic Extraction and Topic Intensity

LDA was first introduced by Blei's study in 2003 (Hofmann, 1999; Blei et al., 2003). Scholars in cell and developmental biology have applied the LDA model to identify scientific research topics (Li et al., 2015; Valle et al., 2020). Besides, perplexity is considered as a standard tool to evaluate the effectiveness of various natural language processing models (Rosen-Zvi et al., 2010). The lower the perplexity, the better the fitting effect of the training topic distribution model to the training set data. Meanwhile, the perplexity would also decrease gradually along with the increase of the topic number. Topic coherence tends to stabilize after reaching the optimal level. When *N* sets to be 20, the title and the abstract topic get their optimum and stabilize. Therefore, we set the number of topics *N* to 20. Figure 1 shows the evaluation of the number of topics in Panel (B). Table 1 lists the 20 topics and their high-frequency keywords.

**Table 1:** Results for topic extraction and topic prediction.

	**Topic name**	**Keywords**	**R2_score (Prophet-logistic, Prophet-liner)**	**Topic %**	**Predicted %**	**Up/Down**
Topic 1	Biomarker discovery in early diagnostics	biomarker, discovery, diagnostics, omics	(0.9542, 0.9982)	3.70	3.21	↓
Topic 2	Machine learning for cancer prediction	prediction, classification, learning, information	(0.9774, 0.9985)	6.40	7.10	↑
Topic 3	Novel biomarker identify technology	profiling, expression, novel, signature	(0.9821, 0.9994)	7.90	9.14	↑
Topic 4	Multi-omics in Hepatocellular Carcinoma	carcinoma, protein, hepatocellular, genomics	(0.9530, 0.9980)	4.20	3.39	↓
Topic 5	Genomics in clinical application	clinical, application, genomics, functional	(0.9434, 0.9956)	3.20	2.81	↓
Topic 6	Genomics in tumor heterogeneity discovery	single, inhibitor, plasma, heterogeneity	(0.9187, 0.9962)	3.30	2.08	↓
Topic 7	Proteomics in post-translational modification	translational, selection, oncology, proteomics	(0.9392, 0.9968)	3.00	2.62	↓
Topic 8	Transcriptional and metabolic processes	pathway, transcriptional, miRNA, metabolic	(0.9571, 0.9975)	4.90	3.80	↓
Topic 9	Metabolomics of breast cancer	metabolomics, breast, colorectal, sequence	(0.9745, 0.9988)	5.20	6.06	↑
Topic 10	Genomics of colorectal cancer	colorectal, pancreatic, genomic, clustering	(0.9836, 0.9992)	9.20	10.10	↑
Topic 11	Multi-omics analysis	genomic, proteomic, metabolomics, multi-omics	(0.9890, 0.9995)	12.90	14.80	↑
Topic 12	Cancer vaccines	clinical, trial, vaccine, alcoholic	(0.9339, 0.9934)	2.70	2.53	↓
Topic 13	Tumor immunotherapy	biology, epidemiology, immunotherapy, regulator	(0.9565, 0.9976)	3.80	3.48	↓
Topic 14	Metabolomics in prostate cancer	prostate, miRNA, metabolic, liver	(0.9065, 0.9924)	2.50	1.63	↓
Topic 15	Pharmacogenomics	medicine, personalize, precision, oncology	(0.9740, 0.9993)	5.40	5.80	↑
Topic 16	Genome-guided therapy regimens	genome, carcinoma, target, treatment	(0.9728, 0.9975)	5.70	5.84	↑
Topic 17	MicroRNAs target genes	breast, target, mediate, microRNA	(0.9788, 0.9989)	6.60	7.65	↑
Topic 18	Microbial metabolomics	mutation, pathway, microenvironment, immune	(0.9299, 0.9945)	3.00	2.26	↓
Topic 19	Metagenomics	environmental, epigenetic, cycle, fingerprinting	(0.9374, 0.9942)	3.10	2.76	↓
Topic 20	Disease gene localization cloning	disease, colon, model, gene	(0.9474, 0.9979)	3.30	2.94	↓

LDAvis an interactive dynamic visualization tool for the LDA model. We use this tool to visualize the results of topic distribution. In Panel (C), the number of circles represents the number of topics, and its size means the corpus belonging to the topic. The quadrants of circle distribution represent the clustering situation. The distance between circles represents the semantic distance between topics. The farther space is, the higher the discrimination between topics is.

Topic intensity is a statistical attribute of topic, indicating the degree of concern of the topic. In our research, we employed the number of documents distributed to each topic to calculate intensity. The intensity index of each topic constitutes a series of time. Define in *e* time slice, in the document set, the number of documents is *Ne*, and the intensity of topic *X*, *T*_*X*_ means the number of articles attributed to topic *X*:

$$\begin{array}{l} Intensity\text{\_}T_{X}=\underset{d\in N_{e}}{\sum }\theta_{dX} \end{array}$$

### Topic Trend Prediction

In our study, we use the time series model, the Prophet neural network, to predict the topic trend. Compared with the classical ARIMA model and LSTM model, the Prophet model can better predict the growth trend. It does not require extensive sample data for text training, so it is easier to achieve convergence than the LSTM method. Since the minimum unit for the journal publication cycle in the data sample is monthly. When predicting the size trend of each topic, we take it monthly, ignoring the influence of cycle factors and holiday factors.

R2_score represents the deviation between the observed value and the real value, ranging from 0 to 1. This is used to evaluate the quality of the trend predictions. The case that R2_score is closer to 1 means the fitting effect is ideal. Besides, we compare Prophet-Liner and Prophet-Logistic results to examine whether the topic trend is linear or not. Intuitively, the Prophet-Liner result represents the direct prediction on linear trend, while the other is the direct prediction on logistic trend.

The ECharts ThemeRriver diagrams are used in the series to represent changes in events or topics over time. The different colored river branches in the theme river encode various topics. The width of the river branch encodes the value in the original dataset. Figure 1 shows the evolution trend for omics data integration in cancer research in Panel (D).

## Results

### Topic Extractions

In Table 1, the results for topic extractions, we only list the top four words with the highest frequency in each topic, excluding terms like “cancer” or “integration” et al., which are less informative in our analysis. In summary, these topics include: biomarker discovery in early diagnostics (86 studies); machine learning for cancer prediction (148 studies); novel biomarker identify technology (183 studies); multi-omics in Hepatocellular Carcinoma (97 studies); genomics in clinical application (74 studies); genomics in tumor heterogeneity discovery (76 studies); proteomics in post-translational modification (70 studies); transcriptional and metabolic processes (114 studies); metabolomics of breast cancer (121 studies); genomics of colorectal cancer (213 studies); multi-omics analysis (299 studies); cancer vaccines (63 studies); tumor immunotherapy (88 studies); metabolomics in prostate cancer (58 studies); pharmacogenomics (125 studies); genome-guided therapy regimens (132 studies); non-coding RNA target genes (153 studies); microbial metabolomics (70 studies); metagenomics (71 studies); genomics of colorectal cancer (77 studies). In Table 1, Column 5 shows the weights of each topic, indicating the numbers of documents that are assigned to the corresponding themes. Topic 11 and Topic 10 hold the most assigned documents, indicating the increased research trends in omics data integration in the multi-omics study.

Column 3 in Table 1 demonstrates the contradistinctive results of two trend prediction methods: The Prophet model (logistic trend) and the Prophet model (linear trend). For each research topic, the R2_score value of the Prophet model exceeds 0.90, indicating that the Prophet model can well fit the evolution trend of the research topic. The reason is that each topic's distribution has a noticeable growth trend, the sequence is non-stationary, so the Prophet model's effect fits well. Besides, most of the research topics are more in line with the Liner trend, for the R2_score is higher than 0.99 on average because most of the topics in this field are in a period of rapid growth and have not yet reached saturation growth.

From the overall development trend of the subject, the research achievements of omics analysis in cancer research increase year by year. Figure 1 Panel (D) shows the result. In recent 5–10 years, omics technology has been widely used. More in-depth exploration has been obtained from various cancer research perspectives, indicating that the integration of omics data has been more recognized by the academic. The application of biomarker discovery technology in the study of cancers is supported by medical and biological research.

### Trend Prediction Analysis

Column 5 in Table 1 shows the predicted results based on the Prophet modified model. It can be seen that the research achievements exhibit a growing trend on the whole. However, the growth trends are slightly different in each topic field. Compared between Column 4 and Column 5 in Table 1, we can see that the weights of Topic 2, 3, 9, 10, 11, 15, 16, 17 are higher than their current proportions. The river scale map of the predicted-modified topic evolution trend also shows the emergence and the development of various topics. Figure 1 Panel (E) shows the topic evolution results in the past 15 years. In terms of published journal articles, these topics maintain a high degree of research interest.

Machine learning is an intelligent scientific tool to improve concrete algorithms in experiential learning (Smith et al., 2020). Machine learning has many cancer applications (Han and Li, 2011; Swan et al., 2013; Kim et al., 2016). It can study tumor subtypes' classification and predict tumor patients' phenotype (Hanczar et al., 2020; Smith et al., 2020). For example, to indicate the treatment effect or to predict the recurrence. Machine learning can be combined with molecular networks to study the molecular mechanisms of tumors (Zhu et al., 2019). The development of tumor genomes has promoted the development of machine learning. Meanwhile, the optimization of machine learning algorithms has also announced the research on phenotypic biomarkers of cancer. Hence, Topic 2 machine learning techniques will be more widely used in study refers to cancer prediction.

Topic 3 and Topic 11 refer to novel biomarker identifies technology and multi-omics analysis. By employing Genomics and proteomics technologies, an immense amount of genomic data is being generated on clinical tumors, which has transformed the cancer landscape and can transform cancer diagnosis and prognosis (Shukla, 2017). The future of metabolomics and other omics approaches rests with their ability to monitor subtle changes that occur before the detection of a gross phenotypic change reflecting disease (Kim et al., 2008, 2015). Triple-negative breast cancer (TNBC) represents ~15% of breast cancers. In light of the complexity of TNBC, by applying transcriptional regulatory and protein-protein interaction networks and tumor necrosis factor signaling pathways can be identified (Karagoz et al., 2015). The statistical clustering approach and the application of the omics methods, both phenotypes, and endotypes, can better illuminate mechanisms and processes that lead to the complexity of asthma (Perlikos et al., 2016). Advances in multi-omics technology have allowed for the delineation of pathways, which will be particularly significant in TH2 low eosinophilic asthma, and also in pauci-inflammatory disease (Abdel-Aziz et al., 2020). Therefore, the multi-omics approach can offer ways forward on novel diagnostics and potentially help to design personalized therapeutics for cancer. In the topic prediction results (see Column 5 and Column 6 in Table 1), there will be more research studies focusing on novel biomarker discovery and multi-omics analysis in the future.

Topic 9 describes the rapid development of the metabolomics approach in breast cancer study. A growing literature reports the use of metabolites to modulate diverse processes, such as stem cell differentiation, oligodendrocyte maturation, insulin signaling, T-cell survival, and macrophage immune responses (Zanni et al., 2017; Guijas et al., 2018; Procopet et al., 2018; Njoku et al., 2020). The links between metabolomics and breast cancer keywords show more than 200 strength links. Metabolomics has enabled researchers to complement genomic and protein level analysis of disease with both semi-quantitative and quantitative metabolite levels, which are the chemical mediators that constitute a given phenotype (Njoku et al., 2020). Breast cancer is associated with significantly lowered plasma aspartate levels in a training group comprising 1:1 breast cancer patients and controls (Xie et al., 2015). Researchers find that lowed circulating aspartate is a crucial metabolic feature of human breast cancer (Huang et al., 2016). Another breakthrough in metabolomics analysis has led to the discovery of new targets for cancer therapy. Unlike genes or proteins, metabolites are often readily available, which means that MAS is broadly amenable to high-throughput screening of virtually any biological system (Guijas et al., 2018). Therefore, increasing clinical research will be discovered by applying metabolomics in breast cancer and other types of cancer diseases.

Topic 10 focuses on the importance of genomics study in colorectal cancer. Colorectal cancer (CRC) is a common and lethal disease with a high therapeutic need. In a range of protein, DNA, and RNA-based biomarkers under investigation for CRC, long non-coding RNAs (lncRNA) plasmacytoma variant translocation have been evaluated as a diagnostic, prognostic, and therapeutic biomarker in colorectal cancer(Ogunwobi et al., 2020). Researchers also identified a unique subclass of colorectal cancer characterized by hypermutation associated with the POLE mutation. 7.2% of Early-onset colorectal cancers (EOCRCs) had the POLE P286R mutation, which was not found in late-onset CRCs (LOCRCs) (Ahn et al., 2016). Biomarker analysis supported the functional equivalence of weekly and every 2nd-week administration of cetuximab. It provided further confirmation that patients with KRAS wild-type metastatic colorectal cancer (mCRC) were those most likely to benefit from cetuximab treatment (Tabernero et al., 2010).

Topic 15 and Topic 16 refer to cancer pharmacogenomics and genome-guided therapy regimens. A key aspect of precision medicine is identifying biomarkers that predict the response to medications (i.e., pharmacogenetics) (Kranzler et al., 2017). The primary pharmacogenomics research strategy is to select candidate genes related to drug absorption, transport, activation, metabolism, initiation, and excretion (Shukla, 2017). Then the relationship between gene variation and drug efficacy is analyzed by statistical principle. Multiple genes with specific types of alterations have now been identified associated with improved response to chemotherapy and radiotherapy (Ostrom et al., 2013). Although many academic achievements on biomarkers have been reported, a few biomarkers are used in cancer drug development and clinical settings (Saito et al., 2013). Thus, to enable the optimal selection of drug(s) for a cancer patient, more research finding for critical biomarkers discovery is of urgent demand.

Topic 17 refers to the MicroRNAs target genes study. MicroRNAs (miRNAs) are short, non-coding RNA that negatively regulates gene expression and are differentially expressed in human cancers (i.e., breast cancer, prostate cancer, lung cancer, or CRC) (Gwak et al., 2014; Li et al., 2016). In the last decade, microRNAs have emerged as a new class of gene regulators. To date, about 2,000 human miRNAs have been reported in miRBase (v22) (Kozomara et al., 2019). By employing an improved algorithm for miRNA target prediction, now miRDB can present transcriptome-wide target prediction data, including 3.5 million predicted targets regulated by 7,000 miRNAs in five species (Chen and Wang, 2020). It has been demonstrated that miRNAs control major cellular processes, including metabolism, developmental timing, stem cell division, cell growth and differentiation, and apoptosis (Hannafon et al., 2011). At both post-transcriptional and translational levels, miRNAs regulate most known protein-coding genes (Konno et al., 2014; Kim and Naisbitt, 2016). Given this expansive role, the discovery of miRNAs contributes to the pathogenesis of many cancer diseases (Xi et al., 2016).

To sum up, the recent development of omics and biomarker discovery technology in the past two decades has brought a burst inflection point in the above fields. The above eight topics will keep a rapid development in the future. Even though the remaining topics trends show decreases, it does not mean the related research are getting less. In Table 1 Column 4, the Prophet-logistic results are lower than Prophet-liner results, indicating that research related to other topics will keep a steady development in the short term.

## Discussion and Conclusion

### Conclusions

The scientific panorama involved in studying the omics data integration toward the mining of biomarkers in cancers is described in the 20 extracted topics. Among the 2,318 sample studies, the core element from the current scientific discussion is the multi-omics analysis. For each topic, the Prophet model can better adapt to the evolution trend. In addition, all of the research topics are in line with the Liner trend, indicating a development stage for omics analysis in cancer studies. The research methodology proposed in this study is hoped to promote a different approach to conceptualizing and treating omics data integration study based on existing methods.

From the perspective of the distance between topics and the evolution of distribution, the innovative technologies of biomarker discovery represented by genomics, metabolomics, and proteomics are the actual contents of integrating omics data in cancer research. Genomics has done extensive research on cancer cells and cancer patients in different treatment stages. With the continuous development and update of international genome-related databases, traditional machine learning algorithms and deep neural networks are essential for data integration and disease prediction. In the future, innovative algorithms will be explored for biomarker discovery technology in different stages of different types of cancer diseases. There will be more research performing clustering optimizations in the development of multi-omics data integration.

Metabolomics has made many achievements in the research of genitourinary tumors, lung cancer, and breast cancer, mainly reflected in the study of metabolic phenotype biomarkers in the metabolic pathway. It is also used in the research of cancer treatment and prognosis prediction. Vertically omics data integration among proteomics, genomics, and metabolomics is becoming more common in the study of cancers. To obtain biological information through omics technology and to discover the molecular mechanism related to cancers are the primary purpose of proteomics research. To detect new marker molecules and to improve the clinical treatment effect, the development of a multi-omics data platform and schema database together with the exploration of a multi-omics feature expression algorithm will have more practical significance.

### Discussions

In omics research toward the mining of biomarkers in cancers, our study summarizes the existing evolution and predicts the future trends of researches. Specifically, the adopted topic model describes and forecasts the change of time segments, which helps track the research development. Additionally, as our results go beyond the original thinking of theory and framework, this study also helps scholars and readers to understand the hot spots and future opportunities regarding omics data integration in cancer studies.

It is noted that there also exist some limitations. On the one hand, although we described the hot research issues and predicted the frontier research issues, there is still a shortage in natural language processing. Due to the variety of text language rules in different stages and different cancer diseases, the same substance can be expounded in several ways. In the process of document-level natural language processing, the expression differences of specific terms cannot be avoided. On the other hand, this paper's conclusion does not involve finding out the research deficiencies, technical challenges, and other omics research problems toward the mining of phenotype-specific biomarkers. Expert systems development and optimizing algorithms will be performed in the next study to provide a more comprehensive analysis of the research problems.

## Data Availability Statement

The original contributions presented in the study are included in the article/supplementary material, further inquiries can be directed to the corresponding author.

## Author Contributions

LN wrote the manuscript. HH contributed to the modification of the manuscript. All authors contributed to the article and approved the submitted version.

## Conflict of Interest

The authors declare that the research was conducted in the absence of any commercial or financial relationships that could be construed as a potential conflict of interest.
